# Development and testing of a four-item version of the physical resilience instrument for older adults (PRIFOR-4)

**DOI:** 10.1016/j.jnha.2024.100250

**Published:** 2024-04-26

**Authors:** Fang-Wen Hu, Yueh-Ping Li, Chia-Ming Chang, Tzu-Yu Lin, Po-Hsuan Lai, Chung-Ying Lin

**Affiliations:** a School of Nursing, College of Nursing, Kaohsiung Medical University, Kaohsiung, Taiwan; b Center for Long-Term Care Research, Kaohsiung Medical University, Kaohsiung, Taiwan; c Department of Medical Research, Kaohsiung Medical University Hospital, Kaohsiung, Taiwan; d Department of Nursing, National Tainan Junior College of Nursing, Tainan, Taiwan; e Department of Geriatrics and Gerontology, National Cheng Kung University Hospital, College of Medicine, National Cheng Kung University, Tainan, Taiwan; f Master Program of Long-Term Care in Aging, College of Nursing, Kaohsiung Medical University, Kaohsiung, Taiwan; g Department of Family Medicine and Community Medicine, E-Da Hospital, I-Shou University, Kaohsiung, Taiwan; h Institute of Allied Health Sciences, College of Medicine, National Cheng Kung University, Tainan, Taiwan; i Biostatistics Consulting Center, National Cheng Kung University Hospital, College of Medicine, National Cheng Kung University, Tainan, Taiwan; j Department of Public Health, National Cheng Kung University Hospital, College of Medicine, National Cheng Kung University, Tainan, Taiwan; k Department of Occupational Therapy, College of Medicine, National Cheng Kung University, Tainan, Taiwan

**Keywords:** Older adults, PRIFOR, Resilience, Short-form, Validity

## Abstract

The 16-item Physical Resilience Instrument for Older Adults (PRIFOR) has good clinimetric properties; however, a shortened PRIFOR would greatly enhance physical resilience measurements in clinical settings. The current analysis aimed to reduce the number of PRIFOR while maintaining its clinimetric properties, emphasizing on its factor structure and convergent validity. A longitudinal study was conducted among 863 patients aged 65 years or older. Four PRIFOR items with high factor loadings were selected to generate the short version of PRIFOR (PRIFOR-4). The PRIFOR-4 was found to have a unidimensional structure (comparative fit index = 0.999; Tucker-Lewis index = 0.998 in the confirmatory factor analysis results) with good convergent validity with various external measures (absolute r = 0.109–0.597; p-values<0.01). Because the PRIFOR-4 contains only four items, the completion time for the respondents reduced three fourths from the original PRIFOR, which may have a marked reduction in the response burden. The PRIFOR-4 is thus an easy-to-use measurement that saves time for healthcare professionals in clinical practice.

## 1 Introduction

The World Health Organization (WHO) has proposed 2021–2030 as the decade of healthy aging and has evolved considerably from the traditional disease treatment approach to delaying or preventing functional decline in older age [1]. In recent years, efforts to preserve function have gained increasing attention in the field of aging research and geriatric medicine. However, maintaining functional ability depends on recovery from injury, illness, or other health stressors that inevitably occur over one’s lifetime [2]. An emerging concept stemming from this work is “physical resilience”, defined as a characteristic that determines an individual’s ability to resist or recover from functional decline following health stressors [3]. Chhetri et al. (2021) postulated a conceptual model showing that the pre-determinants of physical resilience, including: age, psychosocial factors, health behaviors, genetics, and diseases. They also hypothesized that the impact of these factors on physical resilience is mediated through the intrinsic capacity (i.e., vitality, locomotion, psychological function, and cognition) [4]. Furthermore, increasing physical resilience has been associated with increased physical activity, quality of life, enhanced recovery from frailty, better physical and rehabilitative outcomes [5,6].

Using rigorous methodological approaches, the Physical Resilience Instrument for Older Adults (PRIFOR), a questionnaire, is developed to assess physical resilience in older adults suffering from acute health stress [7]. Prior clinimetric evidence of PRIFOR in older patients supported a three-factor structure (positive thinking, hope and adjusting lifestyle, and belief and hopeful mindset) and revealed good reliability and validity [[7], [8], [9]]. Recently, healthcare professionals require quick and reliable measurements to relieve pressure. A shorter version of PRIFOR would greatly enhance the feasibility of measuring activation in an acute clinical setting and would reduce the burden and cost associated with the survey. Therefore, this study aimed to develop and test a short version of PRIFOR with similar clinimetric properties to the original PRIFOR in hospitalized older patients.

## 2 Methods

### 2.1 Study design and participants

We conducted a longitudinal study and identified potential participants from the adult wards of a 1,343-bed tertiary-care medical center in Taiwan. Patients were eligible for inclusion if they were 65 years or older and were able to communicate independently with the researchers. The exclusion criteria were admission due to severe acute illness (immediately requiring intensive care) or the need for hospice care. The participants provided written informed consent and the study was approved by the Institutional Review Board of the participating hospital (IRB No. A-ER-109-233).

### 2.2 Measurements

1 Physical Resilience Instrument for Older Adults (PRIFOR)

PRIFOR is an instrument used to assess physical resilience in older adults. The self-administered PRIFOR consists of 16 items rated on a 5-point Likert scale (1 = strongly disagree, 5 = strongly agree). A high PRIFOR score indicated high physical resilience [7]. The internal consistency of PRIFOR was determined by a Cronbach’s alpha of 0.94, and good validity, known-group validity, concurrent validity, and predictive validity have also been demonstrated [[7], [8], [9]].

2 Cumulative Illness Rating Scale for Geriatrics (CIRS-G)

The CIRS-G assesses the severity of coexisting diseases in 14 organ systems/scales based on the CIRS-G comorbidity index. Severity was scored from 0 (not comorbid) to 4 (highest comorbidity risk). The final score ranged from 0 to 56 [10]. The CIRS-G score is a predictor of mortality after discharge [11].

3 Short Portable Mental Status Questionnaire (SPMSQ)

The SPMSQ contains ten questions that measure the cognitive function of older adults, with each incorrect answer scored as one point. Intact mental function was indicated by fewer than two errors, and scoring was adjusted for education level [12]. The Cronbach’s alpha for the Chinese version of the SPMSQ was 0.70 [13]. Haglund and Schuckid (1976) examined the correlation between SPMSQ scores and the diagnosis of brain damage or dysfunction and the results showed a criterion validity of 0.63 [14].

4 Short Form Mini Nutritional Assessment (MNA-SF)

The MNA is composed of 18 items, which limits its usefulness in screening [15]. Rubenstein et al. (2001) developed a six-item MNA-SF total score ranging from 0 to 14, and scoring for each part categorizes patients in the following manner: normal [[12], [13], [14]], at risk of malnutrition [[8], [9], [10], [11]], or malnourished (0–7). The MNA-SF and MNA scores were strongly correlated (r = 0.945), with a sensitivity of 0.97, a specificity of 1.00, and a diagnostic accuracy of 0.98 for predicting undernutrition [16].

5 5-item Geriatric Depression Scale (GDS-5)

Depressive symptom severity was assessed using the GDS-5. It consists of five items with a binary response scale, with responses to the first item rated as 0 = yes and 1 = no and responses to the following four items rated as 0 = no and 1 = yes. A high score on the GDS-5 indicates high severity of depressive symptoms [17]. It had satisfactory internal consistency (α = 0.83–0.90) and had a sensitivity of 0.94, specificity of 0.81, positive predictive value of 0.81, negative predictive value of 0.94, positive likelihood ratio of 4.92, and negative likelihood ratio of 0.07 [18,19].

6 Katz Index of Independence in Activities of Daily Living (Katz ADL)

The Katz ADL is an instrument used to assess the living ability of older adults. The self-administered Katz ADL consists of seven items rated on a 3-point Likert-type response scale (0 = totally dependent; 2 = independent). A high Katz ADL score indicates high living functional status [20].

7 European QOL-5 Dimensions (EQ-5D) questionnaire

The EQ5D is a 2-part instrument. Part 1 consists of five self-administered items for which different descriptors are used to indicate the levels of health states. Each item was coded into three levels: no health problems (score 1), moderate health problems (score 2), and severe health problems (score 3). The time-trade-off technique was used to merge the scores of the five items for conversion to a 0–1 scale. A high score on the EQ5D indicates a high quality of life. Part 2 of the questionnaire recorded the subject’s self-assessed VAS rating of health on a vertical 20 cm line on which the best and worst imaginable health states scored 100 and 0, respectively [21].

8 Clinical Frailty Scale (CFS)

The CFS, originally published in 2005, contains seven descriptions representing different levels of frailty [22]. Later, the CFS was expanded from a seven-point scale in 2007 to the present nine-point scale. Patients were graded on a scale of 1–9 (1 = very fit; 2 = well; 3 = managing well; 4 = vulnerable; 5 = mildly frail; 6 = moderately frail; 7 = severely frail; 8 = very severely frail; and 9 = terminally ill). Preliminary evidence for the good psychometric properties of the CFS has been demonstrated [23].

The aforementioned instruments were used to examine convergent validity of the shortened PRIFOR because prior evidence shows that physical resilience is associated with comorbidity risk, cognitive function, malnutrition status, depression condition, ADL performance, quality of life, and frailty [5,6].

### 2.3 Data collection

Eligible patients were recruited within 48 h of admission. After obtaining consent, medical records were reviewed and participating patients were interviewed face-to-face at the bedside by a trained research nurse. Medical records provided demographic factors (i.e., age, sex, educational level, and marital status) and the CIRS-G. Patient interviews were conducted to measure health performance on admission (i.e., SPMSQ, MNA-SF, GDS-SF, Katz ADL, PRIFOR, and EQ5D). Follow-up variables were collected from patient interviews at discharge (i.e., CFS and PRIFOR), one month and three months after discharge (i.e., CFS, Katz ADL, and EQ5D).

### 2.4 Data analysis

After using descriptive statistics to summarise patient characteristics (including demographic factors and health performance at admission, discharge, one month and three months after discharge), confirmatory factor analysis (CFA) was conducted using the diagonally weighted least squares (DWLS) estimator. A three-factor structure (positive thinking [PRIFOR items 1–4], coping and adjusting lifestyle [PRIFOR items 5–10], and belief and hopeful mindset [PRIFOR items 11–16]) was tested for PRIFOR using admission and discharge data separately. To develop a short version of PRIFOR (i.e., PRIFOR-4), items with the highest factor loading in each factor were selected to form the PRIFOR-4.

After PRIFOR-4 was developed, CFA with the DWLS estimator was used again to examine whether the selected items could construct a unidimensional structure of physical resilience for admission and discharge data separately. Several fit indices were used to check the data-model fit in the CFA models: comparative fit index (CFI) > 0.9, Tucker-Lewis index (TLI) > 0.9, root mean square error of approximation (RMSEA) < 0.08, and standardised root mean square residual (SRMR) < 0.08. In addition, PRIFOR-4 was examined for item properties of normality (using skewness and kurtosis ranging between −1 and 1), factor loadings from the CFA, and corrected item-to-total correlations. Cronbach’s α was also tested for the PRIFOR-4 to examine its internal consistency.

Lastly, convergent validity of the PRIFOR-4 was tested with the use of the following external measures: CIRG-S (admission), SPMSQ (admission), MNA-SF (admission), GDS-5 (admission), Katz ADL (admission, discharge, one month after discharge, and three months after discharge), EQ5D utility and VAS scores (admission, one month after discharge, and three months after discharge), and CFS (discharge, one month after discharge, and three months after discharge). All convergent validities were examined using Pearson correlations. All analyses were conducted using IBM SPSS software (version 20.0; IBM Corp., Armonk, NY, USA), except for the CFAs, which were analysed using the lavaan package [24] in R software version 4.2.1.

## 3 Results

The mean age of the 863 older patients was 75.17 (SD = 6.74; range = 65−97) years. The sex distribution was relatively balanced (425 [49.2%] males and 438 [50.8%] females), and most patients had completed primary school education (n = 390; 45.2%). Most older patients were married (n = 687; 79.6%), and reported health status from hospital admission, discharge, one month after discharge, and three months after discharge are presented in Appendix 1.

The CFA fit indices indicated that PRIFOR had a satisfactory three-factor structure at admission (CFI = 0.992, TLI = 0.990, RMSEA = 0.043, and SRMR = 0.057) and at discharge (CFI = 0.995, TLI = 0.994, RMSEA = 0.033, and SRMR = 0.058). Appendix 2 presents the factor loadings for PRIFOR. PRIFOR item 2 had the highest factor loadings for both admission (0.836) and discharge (0.812) in the CFA model in terms of positive thinking; PRIFOR item 10 had the highest factor loadings for both admission (0.831) and discharge (0.818) in the factors of cope and adjusted lifestyle. For the factor of Belief and hopeful mindset, PRIFOR item 12 had the highest factor loading at admission (0.842) and the second highest loading at discharge (0.793); PRIFOR item 14 had the second highest factor loading at admission (0.823) and the highest loading at discharge (0.814). As a result, four items (i.e., items 2, 10, 12, and 14) were selected to form PRIFOR-4.

CFA was conducted to examine whether the four PRIFOR items could be integrated into a single latent concept of physical resilience. The one-factor structure of the four items was satisfactory for both admission (CFI = 0.999, TLI = 0.998, RMSEA = 0.022, and SRMR = 0.021) and discharge data (CFI = 0.997, TLI = 0.991, RMSEA = 0.046, and SRMR = 0.032). Moreover, the four PRIFOR items showed adequate item properties in normality (skewness = −0.46 to −0.03 and kurtosis = −0.38 to −0.14 at admission; skewness = −0.51 to −0.17 and kurtosis = −0.25 to 0.19 at discharge), factor loadings (0.716 to 0.815 at admission; 0.734 to 0.773 at discharge), and corrected item-to-total correlations (0.774 to 0.811 at admission; 0.790 to 0.807 at discharge) (Table 1).

**Table 1:** Item properties and scale properties of a four-item version of the physical resilience instrument for older adults (PRIFOR-4).

Item properties	Item #			
	2	10	12	14
Baseline (n = 863)				
Mean (SD)	3.45 (0.72)	3.64 (0.88)	3.43 (0.77)	3.38 (0.79)
Skewness	−0.36	−0.46	−0.38	−0.03
Kurtosis	−0.38	−0.30	−0.14	−0.29
Factor loading	0.751	0.716	0.815	0.737
CITC	0.795	0.811	0.774	0.801
Discharge (n = 607)				
Mean (SD)	3.78 (0.69)	3.85 (0.79)	3.64 (0.67)	3.58 (0.78)
Skewness	−0.35	−0.51	−0.38	−0.17
Kurtosis	0.19	0.06	0.09	−0.25
Factor loading	0.741	0.773	0.734	0.771
CITC	0.800	0.790	0.807	0.794
Scale properties	Baseline	(n = 863)	Discharge	(n = 607)
Cronbach’s α	0.838		0.841	
CFA fit statistics				
χ^2^ (df)	2.85 (2)		4.52 (2)	
Comparative fit index	0.999		0.997	
Tucker-Lewis index	0.998		0.991	
RMSEA	0.022		0.046	
SRMR	0.021		0.032	

Abbreviations: CITC, corrected item-to-total correlation; CFA, confirmatory factor analysis; RMSEA, root mean square error of approximation; SRMR, standardized root mean square residual.

PRIFOR-4 was highly correlated with original PRIFOR (r = 0.917 between PRIFOR-4 and PRIFOR at admission; r = 0.922 at discharge). Moreover, PRIFOR-4 was significantly associated with all external measures (absolute r = 0.109–0.457 at admission and = 0.203–0.597 at discharge). The magnitudes of the associations between PRIFOR-4 and external measures were similar to those of the associations between PRIFOR and external measures (absolute r = 0.142–0.508 at admission; = 0.188–0.696) (Table 2).

**Table 2:** Convergent validity of a four-item version of the physical resilience instrument for older adults (PRIFOR-4).

External measures	Baseline		Discharge	
	PRIFOR-4	PRIFOR	PRIFOR-4	PRIFOR
CIRS-G	−0.109 (0.001)/863	−0.142 (<0.001)/863	−0.203 (<0.001)/607	−0.188 (<0.001)/607
SPMSQ	0.344 (<0.001)/863	0.380 (<0.001)/863	0.390 (<0.001)/607	0.391 (<0.001)/607
MNA-SF	0.308 (<0.001)/689	0.300 (<0.001)/689	0.353 (<0.001)/444	0.339 (<0.001)/444
GDS-5	−0.457 (<0.001)/863	−0.369 (<0.001)/863	−0.380 (<0.001)/607	−0.307 (<0.001)/607
Katz ADL (baseline)	0.240 (<0.001)/863	0.290 (<0.001)/863	0.342 (<0.001)/607	0.333 (<0.001)/607
Katz ADL (discharge)	0.298 (<0.001)/676	0.324 (<0.001)/676	0.446 (<0.001)/444	0.392 (<0.001)/444
Katz ADL (one month after discharge)	0.290 (<0.001)/794	0.295 (<0.001)/794	0.508 (<0.001)/606	0.450 (<0.001)/606
Katz ADL (three months after discharge)	0.267 (0.007)/101	0.300 (0.002)/101	0.280 (0.005)/101	0.248 (0.012)/101
PRIFOR (baseline)	0.917 (<0.001)/863	--	0.782 (<0.001)/607	0.829 (<0.001)/607
PRIFOR (discharge)	0.726 (<0.001)/607	0.829 (<0.001)/607	0.922 (<0.001)/607	--
EQ5D (baseline)	0.494 (<0.001)/374	0.508 (<0.001)/374	0.447 (<0.001)/163	0.429 (<0.001)/163
EQ5D-VAS (baseline)	0.254 (<0.001)/367	0.246 (<0.001)/367	0.530 (<0.001)/163	0.565 (<0.001)/163
EQ5D (one month after discharge)	0.369 (<0.001)/345	0.370 (<0.001)/345	0.419 (<0.001)/163	0.459 (<0.001)/163
EQ5D-VAS (one month after discharge)	0.274 (<0.001)/332	0.283 (<0.001)/332	0.597 (<0.001)/163	0.696 (<0.001)/163
EQ5D (three months after discharge)	0.279 (0.005)/101	0.342 (0.001)/101	0.361 (<0.001)/101	0.366 (<0.001)/101
EQ5D-VAS (three months after discharge)	0.351 (<0.001)/101	0.430 (<0.001)/101	0.515 (<0.001)/101	0.582 (<0.001)/101
CFS (discharge)	−0.272 (<0.001)/863	−0.330 (<0.001)/863	−0.365 (<0.001)/607	−0.389 (<0.001)/607
CFS (one month after discharge)	−0.308 (<0.001)/839	−0.341 (<0.001)/839	−0.395 (<0.001)/607	−0.396 (<0.001)/607
CFS (three months after discharge)	−0.306 (<0.001)/732	−0.307 (<0.001)/732	−0.505 (<0.001)/545	−0.472 (<0.001)/ 545

Note: Values are reported using Pearson’s r (p-value)/n; all correlations were significant.

Abbreviations: CIRS-G, Cumulative Illness Rating Scale-Geriatric; SPMSQ, Short Portable Mental Status Questionnaire; MNA-SF, Short Form Mini Nutritional Assessment; GDS-5, 5-item Geriatric Depression Scale; Katz ADL, Katz Index of Independence in Activities of Daily Living; PRIFOR, Physical Resilience Instrument for Older Adults; EQ5D, EuroQoL 5-dimension questionnaire including both utility and visual analogue scale (VAS) scores; CFS, Clinical Frailty Scale.

## 4 Discussion

Using the CFA factor loadings on PRIFOR, four items were selected to form a short version of PRIFOR (i.e., PRIFOR-4). The four items were chosen because of their high factor loadings in their embedded latent constructs (including positive thinking, Cope and an adjusted lifestyle, and belief and a hopeful mindset). Moreover, the four items satisfactorily covered all the concepts needed for physical resilience as defined by the original PRIFOR [7]. Apart from covering all physical resilience concepts, the four items showed satisfactory item properties with normal distributions and highly corrected item-to-total correlations. The four items agreed well, forming a brief scale for assessing physical resilience. Specifically, the CFA results supported the unidimensionality of PRIFOR-4, which indicates its good convergent validity of PRIFOR-4 (with the CIRS-G, SPMSQ, MNA-SF, GDS-5, Katz ADL, EQ5D, and CFS scores). High correlations were also observed between PRIFOR-4 and original PRIFOR (r > 0.7), indicating satisfactory criterion-related validity.

PRIFOR-4 contains all the underlying factors (including positive thinking, cope and lifestyle adjustment, and belief and hopeful mindset) from the original PRIFOR to illustrate the concept of physical resilience. These three underlying factors correspond to the findings from a previous qualitative study. When older adults experience health stressors, they develop management strategies to facilitate recovery, which manifest in three themes: [1] making flexible adjustments [2], using adequate support systems, and [3] adopting positive thinking [25]. PRIFOR-4 is also similar to characteristics associated with successful physical aging, such as humor, social support, adaptability, and capitalizing on one’s strengths [26]. Furthermore, our study provides evidence that PRIFOR-4 is indeed unidimensional, with all items loaded strongly onto the concept of physical resilience. The unidimensionality demonstrated here provides empirical support for the subdomains of physical resilience embedded in the overall PRIFOR-4 item list.

Chhetri et al. (2021) postulated a conceptual model hypothesized that intrinsic capacity can serve as a determinant of physical resilience from multiple-domain measurements (i.e., SPMSQ, MNA-SF, GDS-5, and Katz ADL) [4,27]. Merchant et al. (2022) indicated that declining physical resilience is considered a marker of accelerated aging and a risk factor for frailty (i.e., CFS) and has been associated with increasing quality of life (i.e., EQ5D) [5,6]. In this study, PRIFOR-4 was significantly associated with the CIRS-G, SPMSQ, MNA-SF, GDS-5, Katz ADL, CFS, and EQ5D scores, indicating good convergent validity of PRIFOR-4.

The PRIFOR-4 is as precise as the 16-item PRIFOR for assessing physical resilience in older patients, with a marked reduction in the burden on respondents. It is an easy-to-use measurement that saves time for healthcare professionals in busy clinical practice. However, this study has several limitations. First, the patients were from the same tertiary care medical center and therefore, our results may have limited generalizability. Second, all patients were able to complete the PRIFOR test; that is, none of them had moderate or severe cognitive impairment. Therefore, our results are not applicable to older patients with moderate or severe cognitive impairment. Third, all external measures used in the present study could only examine convergent validity of the PRIFOR-4; therefore, the evidence of divergent validity of the PRIFOR-4 is yet known. Fourth, physical resilience emphasized on the reserve and stability of physiological systems, further studies would be valuable to examine the association between PRIFOR-4 and physiological/physical indicators (i.e., VO²max or isokinetic strength), immune or metabolic resilience. Fifth, the impact of PRIFOR-4 with clinical outcomes is unexamined. Future studies may want to explore the predictive validity of the PRIFOR-4. Finally, PRIFOR-4 is a subjective measure similar to patient-reported outcomes for assessing physical resilience in older adults, and may be a promising alternative. Although the PRIFOR-4 may only reflect patients’ perspectives of physical resilience, it is also important and has been suggested by the proceedings of the American Geriatrics Society and the National Institute on Ageing State of Resilience Science Conference [28].

In conclusion, evaluating the physical resilience of older patients can help identify high-risk individuals who may not recover well after facing a stressor. Using the PRIFOR-4, we can quickly assess the physical resilience status of older adults. Such endeavors may help healthcare professionals because predictors of vulnerability can inform appropriate and timely treatment selection for patients, rehabilitation strategies to optimize outcomes, and more robust transitional care plans for older patients.

## Author contributions

Fang-Wen Hu and Chung-Ying Lin conceptualized the study. Yueh-Ping Li, Chia-Ming Chang, Tzu-Yu Lin, Po-Hsuan Lai, Fang-Wen Hu, and Chung-Ying Lin were responsible for analysis and interpretation of results. Fang-Wen Hu, Po-Hsuan Lai and Chung-Ying Lin contributed substantially to the writing of the manuscript.

## Conflict of interest

None of the authors have conflicts related to this manuscript.

## Financial disclosure

This study was supported by a grant from the National Science and Technology Council (NSTC 112-2314-B-037-132-MY3).

## Sponsor role

None.

## Figures and Tables

**Table:** 

Characteristics	Mean (SD)/range (n)
Demographics factors	
Age	75.17 (6.74)/65−97 (863)
Gender^a^	
Male	425 (49.2)
Female	438 (50.8)
Educational level^a^	
Illiterate	122 (14.1)
Primary school	390 (45.2)
Junior high school	98 (11.4)
Senior high school	150 (17.4)
College or above	103 (11.9)
Marital status^a^	
Single	18 (2.1)
Married or cohabitated	687 (79.6)
Divorced or widowed	154 (17.8)
Others	4 (0.5)
Health performance at admission	
CIRS-G	7.32 (3.79)/0−22 (863)
SPMSQ	8.66 (1.94)/0−10 (863)
MNA-SF	11.26 (1.92)/0−13 (689)
GDS-5	0.54 (1.07)/0−5 (863)
Katz ADL	9.98 (3.45)/0−14 (863)
PRIFOR	68.59 (9.91)/31−95 (863)
EQ5D	0.79 (0.19)/0.09−1.00 (374)
EQ5D-VAS	61.92 (19.15)/0−100 (367)
Health performance at discharge	
CFS	4.97 (1.08)/2−8 (863)
PRIFOR	72.26 (9.46)/36−95 (607)
Health performance at one month after discharge	
CFS	4.33 (1.15)/1−9 (839)
Katz ADL	12.36 (2.62)/0−14 (794)
EQ5D	0.88 (0.16)/0.18−1.00 (345)
EQ5D-VAS	69.82 (16.94)/0−100 (332)
Health performance at three months after discharge	
CFS	3.77 (1.17)/1−8 (732)
Katz ADL	12.80 (1.91)/6−14 (101)
EQ5D	0.90 (0.14)/0.46−1.00 (101)
EQ5D-VAS	74.10 (13.51)/30−96 (101)

**Table:** 

Factor	Factor loading	
Item # and item description	Baseline	Discharge
Positive thinking		
1. I am able to recover from illness or injury in the expected duration	0.820	0.771
*2. I believe I can recover from every illness or injury*	*0.836*	*0.812*
3. I try to look on the bright side when I am facing illness or injury	0.816	0.800
4. I focus on my remaining abilities, not on what I cannot do	0.789	0.793
Cope and adjust lifestyle		
5. I can cope with the change in my life after illness or injury	0.812	0.779
6. I adjust my way of life after illness or injury	0.783	0.751
7. When I am ill or injured, I accept help from my families and friends	0.505	0.493
8. When I need to, I can find someone to help	0.561	0.556
9. I believe I can handle my daily activities	0.806	0.775
*10. I believe I can recover to do my daily activities after illness or* *injury*	*0.831*	*0.818*
Belief and hopeful mindset		
11. No matter the good or bad things, I believe most of them happen for a reason	0.698	0.733
*12. Past experience gives me confidence to face new challenges and* *difficulties*	*0.842*	*0.793*
13. I am a strong person when I am facing illness or injury	0.735	0.781
*14. I expect and plan for my future life*	*0.823*	*0.814*
15. I can deal with unpleasant or painful feelings like sadness, fear, and anger	0.789	0.790
16. I feel I can handle my life	0.795	0.782

## Appendix 1 Participant characteristics (N = 863)

Notes: ^a^Reported in n (%).

Abbreviations: CIRS-G, Cumulative Illness Rating Scale-Geriatric; SPMSQ, Short Portable Mental Status Questionnaire; MNA-SF, Short Form Mini Nutritional Assessment; GDS-5, 5-item Geriatric Depression Scale; Katz ADL, Katz Index of Independence in Activities of Daily Living; PRIFOR, Physical Resilience Instrument for Older Adults; EQ5D, EuroQoL 5-dimension questionnaire including both utility and visual analogue scale (VAS) scores; CFS, Clinical Frailty Scale.

## Appendix 2 Factor loadings of the physical resilience instrument for older adults (PRIFOR)

Note: Items for PRIFOR-4 are in *italics.* Factor loadings were derived using confirmatory factor analysis (CFA).

Fit statistics of CFA for baseline: χ^2^ (df) = 259.04 (101), comparative fit index = 0.992, Tucker-Lewis index = 0.990, root mean square error of approximation = 0.043, standardized root mean square residual = 0.057.

Fit statistics of CFA for discharge: χ^2^ (df) = 168.44 (101), comparative fit index = 0.995, Tucker-Lewis index = 0.994, root mean square error of approximation = 0.033, standardized root mean square residual = 0.058.

## References

[1] World Health Organization. World report on ageing and health, https://apps.who.int/iris/handle/10665/186463 [accessed 5 May 2023].

[2] Hadley E.C., Kuchel G.A., Newman A.B., Workshop Speakers and Participants (2017). Report: NIA workshop on measures of physiologic resiliencies in human aging. J Gerontol A Biol Sci Med Sci.

[3] Whitson H.E., Duan-Porter W., Schmader K.E., Morey M.C., Cohen H.J., Colón-Emeric C.S. (2016). Physical resilience in older adults: systematic review and development of an emerging construct. J Gerontol A Biol Sci Med Sci.

[4] Chhetri J.K., Xue Q.L., Ma L., Chan P., Varadhan R. (2021). Intrinsic capacity as a determinant of physical resilience in older adults. J Nutr Health Aging.

[5] Merchant R.A., Izquierdo M., Woo J., Morley J.E. (2022). Resilience and the future. J Frailty Aging.

[6] Hu F.W., Yueh F.R., Fang T.J., Chang C.M., Lin C.Y. (2024). Testing a conceptual model of physiologic reserve, intrinsic capacity, and physical resilience in hospitalized older patients: a structural equation modelling. Gerontology.

[7] Hu F.W., Lin C.H., Yueh F.R., Lo Y.T., Lin C.Y. (2022). Development and psychometric evaluation of the Physical Resilience Instrument for Older Adults (PRIFOR). BMC geriatr.

[8] Hu F.W., Lin C.H., Lai P.H., Lin C.Y. (2021). Predictive validity of the Physical Resilience Instrument for Older Adults (PRIFOR). J Nutr Health Aging.

[9] Lin C.Y., Ou S.H., Chang C.M., Hu F.W. (2023). The Physical Resilience Instrument for Older Adults (PRIFOR) in surgical inpatients: further evidence for its factor structure and validity. J Frailty Aging.

[10] Miller M., Towers A. (1991).

[11] Soh C.H., Ul Hassan S.W., Sacre J., Maier A.B. (2020). Morbidity measures predicting mortality in inpatients: a systematic review. J Am Med Dir Assoc.

[12] Pfeiffer E. (1975). A short portable mental status questionnaire for the assessment of organic brain deficit in elderly patients. J Am Geriatr Soc.

[13] Chou K.L. (2002). Hong Kong Chinese everyday competence scale: a validation study. Clin Gerontol.

[14] Haglund R.M., Schuckit M.A. (1976). A clinical comparison of tests of organicity in elderly patients. J Gerontol.

[15] Vellas B., Guigoz Y., Garry P.J., Nourhashemi F., Bennahum D., Lauque S. (1999). The Mini Nutritional Assessment (MNA) and its use in grading the Nutritional state of elderly patients. Nutrition.

[16] Rubenstein L.Z., Harker J.O., Salvà A., Guigoz Y., Vellas B. (2001). Screening for undernutrition in geriatric practice: developing the short-form mini-nutritional assessment (MNA-SF). J Gerontol A Biol Sci Med Sci.

[17] Hoyl M.T., Alessi C.A., Harker J.O., Josephson K.R., Pietruszka F.M., Koelfgen M. (1999). Development and testing of a five-item version of the Geriatric Depression Scale. J Am Geriatr Soc.

[18] Rinaldi P., Mecocci P., Benedetti C., Ercolani S., Bregnocchi M., Menculini G. (2003). Validation of the five-item geriatric depression scale in elderly subjects in three different settings. J Am Geriatr Soc.

[19] Li Y.P., Lin C.Y., Hu F.W., Shih S.A. (2021). Short versions of the Geriatric Depression Scale (GDS) among widowed older people in Taiwan: comparing their psychometric properties. Australas J Ageing.

[20] Hughes S.L., Edelman P.L., Singer R.H., Chang R.W. (1993). Joint impairment and self-reported disability in elderly persons. J Gerontol.

[21] EuroQol Group (1990). EuroQol--a new facility for the measurement of health-related quality of life. Health Policy.

[22] Rockwood K., Song X., MacKnight C., Bergman H., Hogan D.B., McDowell I. (2005). A global clinical measure of fitness and frailty in elderly people. CMAJ.

[23] Pulok M.H., Theou O., van der Valk A.M., Rockwood K. (2020). The role of illness acuity on the association between frailty and mortality in emergency department patients referred to internal medicine. Age Ageing.

[24] Rosseel Y. (2012). Lavaan: an R package for structural equation modeling. J Stat Soft.

[25] Yueh F.R., Pan J.H., Lee H.F., Yen M., Hu F.W. (2023). A qualitative exploration of older patients’ experiences with frailty and related management strategies. J Nurs Res.

[26] Resnick B., Gwyther L.P., Roberto K.A. (2020).

[27] Chen Y.J., Kukreti S., Yang H.L., Liu C.C., Yeh Y.C., Fung X.C.C. (2023). Psychometric properties of instruments assessing intrinsic capacity: a systematic review. Asian J Soc Health Behav.

[28] Abadir P.M., Bandeen-Roche K., Bergeman C., Bennett D., Davis D., Kind A. (2023). An overview of the Resilience world: Proceedings of the American Geriatrics Society and National Institute on Aging State of Resilience Science Conference. J Am Geriatr Soc.

